# Low Soluble Programmed Cell Death Protein 1 Levels After Allogeneic Stem Cell Transplantation Predict Moderate or Severe Chronic GvHD and Inferior Overall Survival

**DOI:** 10.3389/fimmu.2021.694843

**Published:** 2021-09-24

**Authors:** Lambros Kordelas, Ulrike Buttkereit, Falko M. Heinemann, Peter A. Horn, Bernd Giebel, Dietrich W. Beelen, H. Christian Reinhardt, Vera Rebmann

**Affiliations:** ^1^ Department of Hematology and Stem Cell Transplantation, University Hospital Essen, Essen, Germany; ^2^ Institute for Transfusion Medicine, University Hospital Essen, Essen, Germany

**Keywords:** allogeneic hematopoietic stem cell transplantation, graft-*versus*-host disease, PD-1, soluble PD-1, immune checkpoint, inhibitory co-receptor, GvHD biomarker

## Abstract

Programmed cell death protein-1 (PD-1) is an inhibitory co-receptor required for regulating immune responsiveness and maintaining immune homeostasis. As PD-1 can be released as bioactive soluble molecule, we investigated the clinical significance of soluble PD-1 (sPD-1) after allogeneic hematopoietic stem cell transplantation (HSCT) regarding graft-versus-host disease (GvHD), relapse, and overall survival (OS) in a mono-centric cohort of 82 patients. Compared to pre-HSCT and to healthy controls, post-HSCT sPD-1 plasma levels were significantly increased during an observation time of three months. Univariate analysis revealed that low sPD-1 plasma levels at month one, two or three post HSCT were associated with acute GvHD grade III-IV, the onset of moderate/severe chronic GvHD (cGvHD) and inferior OS, DFS, and TRM, respectively. No relationship was detected to relapse rates. sPD-1 plasma levels were significantly increased in ATG-treated patients compared to ATG-untreated patients. Multivariate analysis revealed that a low sPD-1 plasma levels status at one or two month(s) after HSCT is an independent indicator for inferior OS, DFS, or TRM. A low sPD-1 plasma levels status at month three post HSCT is predictive for the onset of moderate/severe cGvHD. Thus, our study pinpoints the soluble inhibitory co-receptor PD-1 as a promising candidate molecule for the prediction of clinical HSCT outcome.

## Introduction

Allogeneic hematopoietic stem cell transplantation (HSCT) is a well-established cellular immunotherapy for a variety of malignant and non-malignant hematological diseases. The outcome of HSCT is determined by a balance of immune tolerance and immune alloreactivity. If the pendulum swings towards excessive immune alloreactivity, graft-versus-host disease (GvHD) may result. If the pendulum swings towards exaggerated immune tolerance, the graft-versus-leukemia (GvL) effect, which is crucial for the prevention of relapse, is abrogated.

Immune checkpoint molecules function as physiological “brakes” of the immune system responsible for immune homeostasis (1). Inhibitory checkpoint ligands expressed on malignant cells enable tumors to escape anti-tumor immune responses. Specifically, the PD-1/PD-L1 signaling pathway can be hijacked as an immune escape mechanism in hematological malignancies (2). Programmed death-1 (PD-1, CD279) is expressed by activated CD4+ and CD8+ T cells, B cells, monocytes, dendritic and NK cells (3). PD-1 interacts with its cognate ligands PD-L1 (CD274) and PD-L2 (CD273). PD-L1 expression is upregulated by various tumor types including hematological malignancies.

With the advent of immune checkpoint inhibitors (ICI), a novel therapeutic modality of cancer immunotherapy has enriched the clinical armamentarium. Initially, ICI were deployed in solid cancers, but they are now increasingly used in hematological diseases and even after allogeneic HSCT. ICI after allogeneic HSCT are associated with the potentially severe or even lethal risk of inducing GvHD (4–6). A recent review summarizes that ICI is associated with GvHD not only if used after allogeneic HSCT, but also ICI prior to allogeneic HSCT is associated with acute GvHD (aGvHD) in 56% and with chronic GvHD (cGvHD) in 29% of patients (7).

There are only a few studies analyzing PD-1 expression after HSCT without checkpoint inhibition. Simonetta et al. (8) observed a significantly increased PD-1 expression on CD4+ and CD8+ T cells in 105 HSCT patients early after HSCT compared to healthy controls (HC). In the later course, the authors describe a progressive normalization of PD-1 expression on CD8+, but not on CD4+ T cells. The authors found no association of PD-1 expression on CD4+ and CD8+ T cells with donor/recipient matching, stem cell source, type of conditioning regimen and donor CMV sero-status. Noteworthy, T cell depletion (TCD) in general was significantly associated with elevated PD-1 expression on both CD4+ and CD8+ T cells. Since in this cohort various methods of in*-* and ex-vivo TCD were applied, the authors could discriminate that in-vivo TCD with ATG was associated with increased PD-1 expression on CD4+, but not on CD8+ T cells. In contrast, ex-vivo TCD with alemtuzumab or *in-vivo* TCD with post-cyclophosphamide were significantly associated with PD-1 upregulation on both CD4+ and CD8+ T cells. The authors conclude that these results might explain different effects of PD-1/PD-L1 blockade and also the associated different risk levels for GvHD depending on the time of administration. According to Jain et al. (9) PD-1 expression was elevated on T cells both in relapsed and non-relapsed patients after HSCT, indicating that membrane-bound PD-1 is not a dominant marker for leukemia-specific T cell exhaustion in the context of post-HSCT relapses.

Notably, PD-1 can be expressed as co-inhibitory receptor on cell surfaces, but it can also be released in soluble forms that can be detected in the plasma of respective patients. Soluble PD-1 is biologically active and can inhibit the interaction of membrane-bound PD-1 with PD-L1 and PD-L2 (10). Since none of the aforementioned studies, report on *soluble* PD-1 (sPD-1), we focused on sPD-1 in our monocentric study and investigated sPD-1 concentrations in plasma samples of 82 HSCT patients before and during the first three months after HSCT.

## Material and Methods

### Study Design

This prospective monocentric study was approved by the Ethical Board of the University Hospital of Essen (07-3503) and conducted in accordance to the Declaration of Helsinki. All patients gave their informed consent to participate in this study. Ethylenediaminetetraacetate (EDTA) plasma samples were serially collected from the patients before as well as 1, 2, and 3 month(s) post transplantation.

### Patients’ HSCT Disease Characteristics and GvHD Classification

Disease stage was classified according to the EBMT risk score for outcome after HSCT (11). Early disease stage included acute leukemia (AL) transplanted patients in first complete remission (CR), myelodysplastic syndrome (MDS) either untreated or in first CR, Non-Hodgkin’s lymphoma (NHL) and multiple myeloma (MM) transplanted patients either untreated or in first CR; intermediate stage included AL in second CR, MDS in second CR or in partial remission (PR), NHL and MM in second CR, in PR or in stable disease. All other disease stages were considered as late stages.

Acute and chronic GvHD was categorized according to accepted standards (12–14). We grouped aGvHD grade 0-II as mild manifestations of aGvHD in contrast to aGvHD III-IV as severe manifestations of aGvHD. Acute and chronic GvHD were categorized according to the NIH 2005 criteria. Accordingly, we classified no or mild cGvHD as minor manifestations of cGvHD and compared these to moderate and severe cGvHD.

### Quantification of Soluble PD-1

The determination of plasma levels of soluble PD-1 (sPD-1) was carried out as previously described (15) using a commercial ELISA kit (DuoSet ELISA Development System DY1086/CD279; R&D Systems, Wiesbaden-Nordenstadt, Germany) with minor modifications. Briefly, microtiter plates with high binding surface (Costar Corning, Bodenheim, Germany) were coated with anti-human PD-1 (842902 R&D Systems) at 4°C overnight at a final concentration of 1 µg/ml. Thereafter, free binding sites were blocked with phosphate-buffered saline (PBS) supplemented with 1% bovine serum albumin (BSA, AppliChem GmbH, Darmstadt, Germany) and 0.05% Tween-20 (Carl Roth GmbH, Karlsruhe, Germany). Plasma samples were used undiluted and tested in duplicate. Recombinant PD-1 protein fused with the Fc-portion of human IgG (R&D Systems) was used as standard reagent and serially diluted in concentrations ranging from 0 to 10,000 pg/ml. Detection reagent of bound PD-1 (842903, R&D Systems) was used in a concentration of 50 ng/ml being diluted in blocking buffer. Bound detection antibodies were recognized by streptavidin conjugated with horseradish peroxidase (R&D Systems) being diluted 1:200 in blocking buffer. The 3,3,5,5-tetramethybenzidine substrate reagent set (Becton Dickinson, Heidelberg, Germany) served as substrate. The substrate reaction was stopped by 2N H2SO4 and optical density was measured at 450 nm (Biotek Instruments, Winooski, VT, USA). Quantification of PD-1 plasma levels was performed by four-parameter curve fitting. Intra- and interassay coefficients of variation were 13.0% and 19%, respectively.

### Flow Cytometry Analysis

Cell surface expression was analyzed by staining with fluorchromes-conjugated mononuclear antibodies against human CD3 (ECD, clone OKT3; Beckman Coulter, Krefeld, Germany) and PD-1 (AF488, clone EH12.2H7, BioLegend, Koblenz, Germany). Isotype-matched antibodies served as negative controls (BD Bioscience, Heidelberg, Germany). Samples were subjected to flow cytometry using a CytoFlexS cytometer (Beckman Coulter). Data acquisition of at least 200,000 events was performed with CytExpert Version 2.1 software (Beckman Coulter) and analyzed with Kaluza Analysis 2.1 software. Mean fluorescence intensity (MFI) index was defined by the ratio obtained from mean intensity of PD-1 staining on CD3 divided by the corresponding isotype matched control.

### Statistics

Statistical analyses and presentation were performed by using SPSS 23.0 (SPSS Inc., Chicago, IL, USA) or GraphPad Prism V8.4.3 software (GraphPad Software, San Diego, CA, USA). Data are presented either as median with range or as mean ± SEM (standard error of mean). After testing for Gaussian distribution, continuous variables were compared by T-test, non-parametric Mann-Whitney or two-way analysis of variance (Kruskal-Wallis test with uncorrected Dunn’s test for multiple comparison), as appropriate. Nonparametric Spearman correlation was used to correlate the sPD-1 levels with PD-1 surface expression on CD3+ T cells. For categorical data, 2-sided Fisher’s exact test was used. Clinical outcome endpoints of the study were overall survival (OS), disease-free survival (DFS), transplant-related mortality (TRM), acute graft-versus-host disease (aGvHD) grade III-IV, and moderate/severe chronic GvHD (cGvHD). OS was defined as time from HSCT to death from any cause. DFS was assessed as time from HSCT to treatment failure due to relapse, whereas TRM was assessed as time from HSCT to death without any sign of relapse. Using BIAS 11.08 software program (http://www.biasonline.de/) receiver operating characteristic (ROC) analysis was performed to define the optimal threshold value for sPD-1 regarding sensitivity and specificity to stratify the continouous parameter into a dichotomous variable for the prediction of aGvHD, cGvHD, OS, DFS, and TRM. Probabilities of OS and DFS were analyzed using the Kaplan-Meier method in combination with the log-rank test implemented in the R package survminer (version 0.4.0; https://CRAN.R-project.or/package=survminer). Stepwise multivariate Cox regression according to proportional hazards assumption was used to identify prognostic factors for OS and DFS, respectively. For moderate/severe cGvHD, aGvHD grade III-IV was defined as competing event. Relapse was the competing risk event for TRM. Univariate competing risk analysis by Aalen-Johanson-estimator and multivariate competing risk regression models were performed using BIAS 11.08 or STAT MP 16.0. Covariates were included into the multivariate analyses based on conceptual evaluation of literature or being associated with a p-value <0.05 to certain clinical parameters in univariate analysis. Statistical significance was defined as p ≤ 0.05.

## Results

### Patient Characteristics

Eighty-two patients, 42 female and 40 male, were enrolled in the study. Median age was 56 years (range 19-75 years). The majority (40 patients [pts.]) were diagnosed with Acute Myeloid Leukemia (AML). Other diagnoses included Myelodysplastic Syndrome (MDS, 8 pts.), Acute Lymphoblastic Leukemia (ALL, 8 pts.), Non-Hodgkin Lymphoma (NHL, 12 pts.), Myeloproliferative Neoplasms (MPN, 12 pts.) and other (2 pts.). These patients underwent HSCT between April 2017 and March 2019 at the Department of Hematology and Stem Cell Transplantation of the University Hospital Essen, Germany. Median CD34+ transplanted was 6.7 x 10^6^/kg body weight (BW) of the recipient (range 3.0-19.5). The patients’ and HSCT characteristics are detailed in **Table 1**. Fifty-six (68%) of the 82 patients received anti-thymocyte globulin (ATG) as *in vivo* T-cell depletion. Thirty-two patients received total body irradiation (TBI) as part of the conditioning regimen. Twenty-one patients received grafts from related donors; the remaining 61 patients received grafts from unrelated donors. In 73 cases the HSCT was HLA-identical, 9 patients were transplanted with an HLA-mismatched graft. Obviously, related vs. unrelated donors and GvHD prophylaxis were significantly different in the ATG-treated compared to the non-ATG-cohort. Median age, also, was significantly higher in the ATG-treated compared to the non-ATG-cohort. Besides, there were no significant difference in gender, diagnoses, CD34+ cells/kg BW, HLA-identical *vs.* mismatched, acute GvHD grade 0-II vs. III-IV, no/mild *vs.* moderate/severe chronic GvHD, relapse and OS when comparing the ATG- and the non-ATG-cohort (**Table 1**). At a median follow-up of 310 days (range: 22-791) after HSCT, 9 patients (11%) had suffered a relapse and 62 patients (76%) were alive.

**Table 1:** Demographic and HSCT characteristics of patients.

Number of patients (N;%)	All patients	Non ATG-treated	ATG-treated*	p-value**
	82	26 (32%)	56 (68%)	
Median age [years(range)]	56 (19-75)	51 (20-69)	59 (19-75)	0.02
Gender (female/male)	42/40	13/13	29/27	n.s.
Diagnosis at allo SCT				n.s.
AML	40	12	28	
MDS	8	1`	7	
ALL	8	3	5	
NHL	12	4	8	
MPN	12	3	9	
Other	2	2	1	
CD34 x 10^6^/kg BW[median(range)]	6.7 (3.0-19.5)	7.1 (3.1-13.3)	6.3 (3.0-15.0)	n.s.
Conditioning				n.s.
TBI(8-12 Gy) & Flu, cycloph or Etopos	32	11	21	
Fludarabine and Busulfan	28	7	21	
Fludarabine and Treosulfane	18	5	13	
Other	4	3	1	
Unrelated donor(URD) yes/no	61/21	7/19	54/2	<0.0001
HLA-identical yes/no	73/9	23/3	50/6	n.s.
Female to male HSCT yes/no	12/70	7/19	5/51	0.0451
Follow-up time [days(median,range)]	310 (22-791)			
GvHD prophylaxis				2
CSA & MTX	66	15	51	
CNI*** & MMF	14	11	3	
Other	2	0	2	
Acute GvHD****				
Onset acute GvHD(median, range)	16 (10-65)	16 (10-31)	16 (10-65)	n.s.
Acute GvHD grade 0-II(max. severity)	74	24	50	
Acute GvHD grade III-IV(max. severity)	8	2	6	n.s.
Chronic GvHD****				
Onset chronic GvHD(median, range)	152 (100-690)	191 (101-690)	142 (100-523)	n.s.
no/mild chronic GvHD(max. severity)	52	15	37	
Moderate/severe chronic GvHD(max. severity)	20	9	11	n.s.
Relapse (yes/no) N (%)	9/73 (11%/89%)	1/25 (1%/30%)	8/48 (10%/59%)	n.s.
Survival (yes/no) N (%)	62/20 (76%/24%)	21/5 (26%/6%)	41/15 (50%/18%)	n.s.

*All but one patient received ATG Neovii™ in a cumulative dosage of 30-60 mg/kg BW. One patient received Thymoglobulin Genzyme™ in a dosage of 6 mg/kg BW.

** Comparisons between patients treated with ATG and non-treated with ATG (Fisher’s exact test or unpaired t-test); n.s., not significant.

***CNI, CSA or Tacrolimus.

****GvHD not evaluated for all patients due to death/missing clinical data. Maximal severity for acute and chronic GvHD are indicated.

The median onset of acute GvHD was 16 days (range: 10-65). In our cohort, all acute GvHD showed classical onset, there were no cases of late onset aGvHD. The distribution of maximal aGvHD was as follows: no aGvHD: 4; aGvHD I°: 53; aGvHD II°: 17; aGvHD III°: 7; aGvHD IV°: 1. Regarding chronic GvHD, 24 patients showed no symptoms of cGvHD; 2 pts. developed *de novo*-cGvHD without prior aGvHD; 23 pts. had quiescent cGvHD; 23 pts. suffered from progressive cGvHD out of aGvHD; data n.a.: 10. The median onset of chronic GvHD was 152 days (range: 100-690). The distribution of maximal cGvHD was as follows: no or mild cGvHD: 52; moderate cGvHD: 9; severe cGvHD: 11; data n.a.: 10. Transplant-related mortality was categorized according to etiology either due to infection, organ toxicity and acute GvHD with the following results: TRM due to infection: 6; TRM due to organ toxicity: 5; TRM due to aGvHD: 6. The other three deaths were due to relapse of the hematological disease.

### Increased Levels of sPD-1 Post HSCT

**Figure 1:**
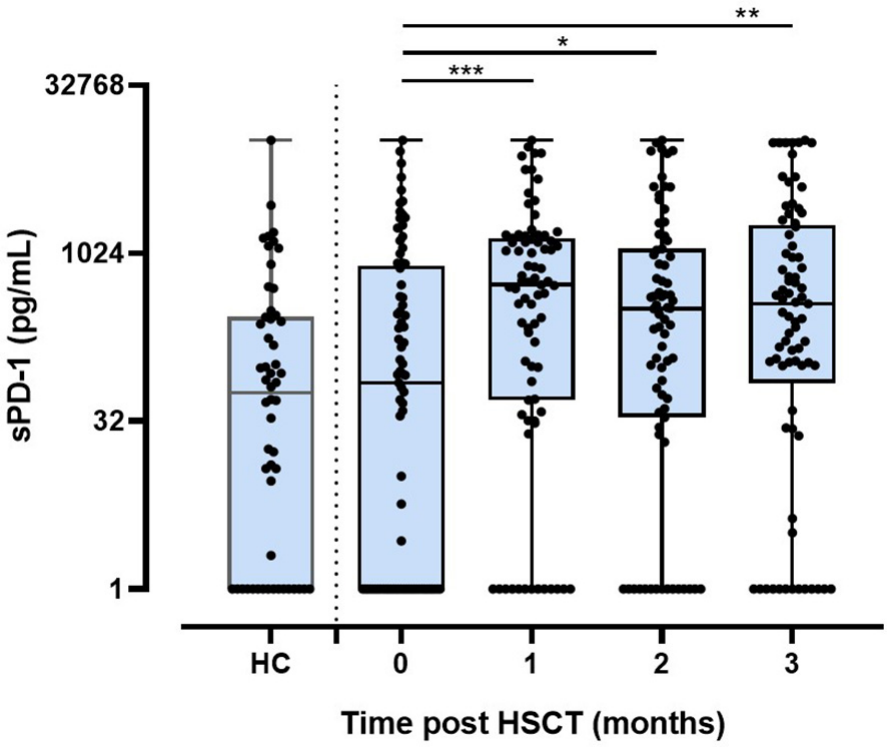
Levels of sPD-1 in healthy controls and in patients before and post HSCT during the first three months. HC, healthy controls (n=54); 0: before HSCT; 1, 2, 3: one, two, three month(s) post HSCT; *p < 0.05,**p < 0.01; ***p < 0.001.

**Figure 2:**
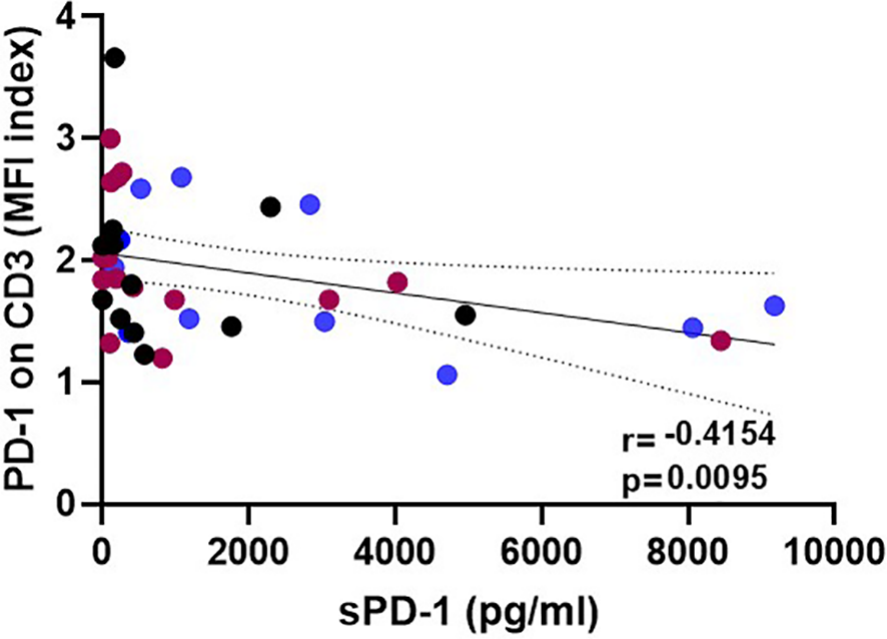
Inverse correlation of post-HSCT sPD-1 levels with surface expression on CD3+ T cells. Plasma samples and corresponding peripheral blood lymphocytes were taken at various time points: blue, brown, and black dots correspond to 1, 2, and 3 month(s) post HSCT, respectively; solid line indicates regression line; dotted lines represent the corresponding 95% confidence bands; r: correlation coefficient value.

To study the effect of HSCT on the release of soluble PD-1 (sPD-1) molecules into the blood circulation, sPD-1 levels were compared among healthy controls (HC) and patients pre-HSCT and post-HSCT. sPD-1 levels of HC were not significantly different from the ones of pre-HSCT patients (**Figure 1**). Furthermore, pre-HSCT sPD-1 levels were not associated to patients’ gender and disease (**Supplementary Figures 1A, B**). However, sPD–1 levels were significantly increased (p<0.001, p<0.5 and p<0.01) one, two and three months post-HSCT, compared to pre-HSCT levels; and overall, no substantial fluctuation of sPD-1 was observed within this observation period (**Figure 1**). Of note, an inverse correlation of post sPD-1 levels (r=-0.4154, p=0.0095) with the PD–1 intensity of cell surface expression on CD3+ T cells was observed in 13 patients post HSCT (**Figure 2**).

### The Course of sPD-1 Levels Are Decreased in Patients With GvHD and Inferior OS, DSF, and TRM

**Figure 3:**
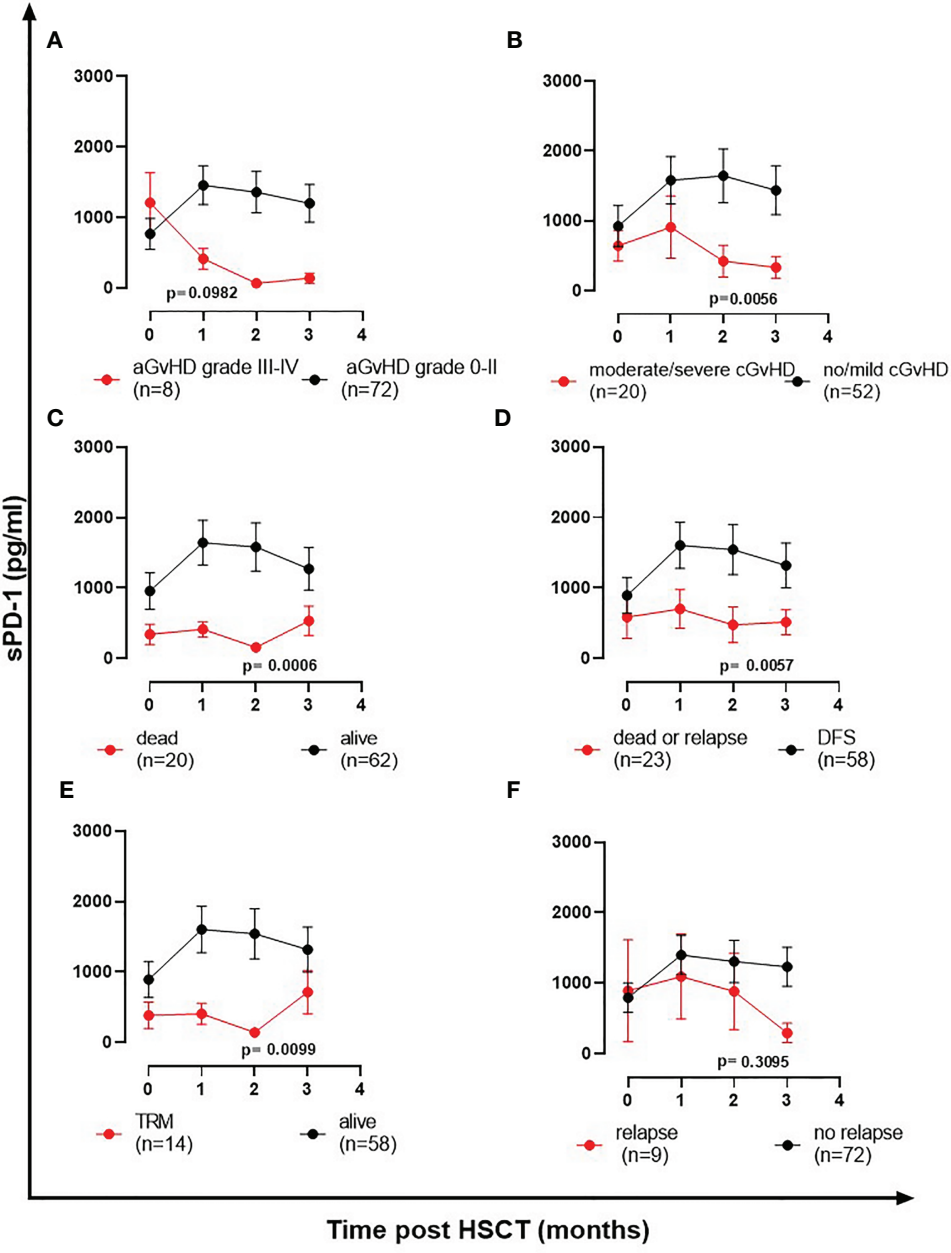
The course of sPD-1 levels in relationship to clinical outcome post HSCT. Course of sPD-1 levels in patients with **(A)** aGvHD grade III-IV (red line) versus aGvHD grade 0-II (black line), **(B)** moderate/severe cGvHD (red line) versus no/mild cGvHD (black line), **(C)** patients passed away (red line) versus patients being alive (black line) at month three, **(D)** patients passed away or with relapse (red line) *versus* patients with disease-free survival (black line), **(E)** patients with transplant-related mortality (TRM) and not due to relapse (red line) versus patients being alive (black line), and **(F)** patients experiencing recurrence (red line) *versus* patients without relapse during the follow-up time. Data are presented as mean ± SEM before HSCT (0) and one, two, three month(s) post HSCT. Manifestation of aGvHD or cGvHD could not be evaluated for all patients due to death or missing clinical data.

To investigate the association of sPD-1 plasma levels with HSCT outcome, the course of sPD-1 levels was related to aGvHD, cGvHD, OS, DFS, TRM, and relapse (**Figure 3**). The sPD-1 levels (mean ± SEM, pg/ml) of 8 patients with severe aGvHD grade III-IV presented decreasing sPD-1 levels within the first three months post HSCT, whereas the sPD-1 levels of patients with aGHD grade 0-II (n=72) were higher during this observation time (**Figure 3A**). Significantly decreased sPD-1 levels (p=0.0056) were observed for 20 patients experiencing moderate or severe cGvHD compared to 52 patients with no or only mild cGvHD (**Figure 3B**). Among patients with moderate/severe cGvHD, the course of sPD-1 levels was not different between quiescient and progressive cGvHD (**Supplementary Figure 2**). Noteworthy, lower sPD-1 levels before and during the obervation time were significantly associated with inferior OS (p=0.0006, **Figure 3C**), DFS (p=0.0057, **Figure 3D**), and TRM (p=0.0099, **Figure 3E**) post HSCT. Even though patients who experienced disease recurrence displayed lower sPD-1 levels two and three months post HSCT compared with patients without a relapse, the course of these patients’ groups appeared not to be significantly different (**Figure 3F**).

### Low sPD-1 Levels at Month Two or Three Post HSCT Are Indicators for aGvHD Grade III-IV and the Onset of Moderate/Severe cGvHD

In order to identify a potential threshold level indicating an increased risk for severe aGvHD grade III-IV, receiver operating characteristic (ROC) analysis was performed (**Table 2**). A significant sPD-1 cut-off level of 461 pg/ml one month post HSCT was defined for the onset of moderate/severe cGvHD, whereas no relevant threshold values could be identified for aGvHD grade III-IV. Using sPD-1 cut-off level of 461 pg/ml, univariate competing risk analysis for moderate/severe cGvHD with aGvHD grade III-IV as competing event did not present statistically different cumulative incidence functions neither for cGvHD (p=0.059) nor for the competing risk aGvHD (p=0.116, **Supplementary Figures 3A, B**).

**Table 2:** sPD-1 cut-off levels defined by ROC analysis for aGvHD, cGvHD, OS, DFS, and TRM at month one, two or three post HSCT.

HSCT endpoint	Month post HSCT	Cut-off	AUC	Sensitivity	Specificity	p
**aGvHD grade III-IV**	1	1229	0.605	34.7	100.0	0.359
	2	133	0.744	64.7	100.0	0.048
	3	405	0.699	46.2	100.0	0.139
**moderate/severe cGvHD**	1	461	0.675	61.6	75.0	0.019
	2	133	0.690	90.0	68.9	0.015
	3	107	0.724	60.0	77.1	0.003
**OS**	1	567	0.655	83.3	57.4	0.045
	2	415	0.709	94.1	52.6	0.008
	3	489	0.517	78.5	41.3	0.841
**DFS**	1	567	0.621	76.2	56.2	0.100
	2	415	0.664	85.0	51.9	0.030
	3	1633	0.543	94.1	25.4	0.590
**TRM**	1	567	0.675	83.3	56.9	0.056
	2	415	0.723	90.1	51.8	0.020
	3	140	0.515	77.8	41.8	0.885

p-values were defined by Mann-Whitney test. AUC, Area under curve; aGvHD, acute Graft-versus-Host Disease; cGvHD, chronic Graft-versus-Host Disease; OS, overall survival; DFS, disease-free survival; TRM, transplantation-related mortality.

**Figure 4:**
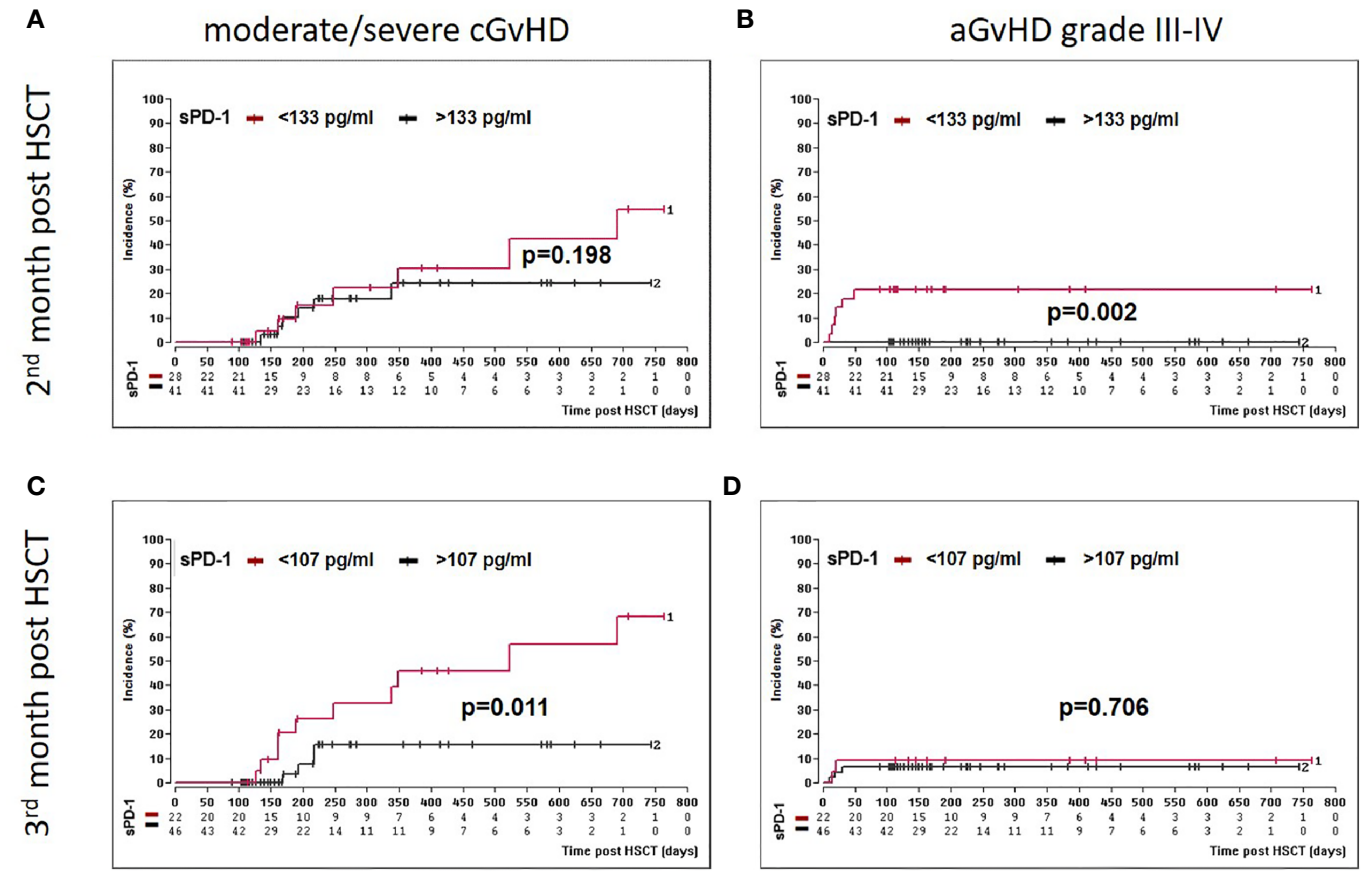
Association of low sPD-1 level with aGvHD grade III-IV and moderate/severe cGvHD. Patients were divided into two groups according to cut-off levels [(< or >133 pg/ml, **(A, B)**] or [(> or <107 pg/ml, **(C, D)**] obtained two **(A, B)** and three months **(C, D)** post HSCT, respectively. Estimated cumulative incidence curves of patients with moderate/severe cGvHD **(A, C)** and aGvHD grade III-IV **(B, D)** as competing event are shown for patients with sPD-1 <133 pg/mL **(A, B)** or <107 pg/ml **(C, D)** in brown and compared to patients with >133 pg/mL(A, B) and >107 pg/ml **(C, D)** in black. The sPD-1 levels were available only for 69 patients **(A, B)** at month two or 68 patients **(B, C)** at month three post HSCT due to death or loss of follow up.

Two months post HSCT, a sPD-1 cut-off level of 133 pg/ml was significantly associated with both, aGvHD grade III-IV and the onset of moderate/severe cGvHD (**Table 2**). Here, univariate competing risk analysis did not present statistically different cumulative incidence functions for cGvHD (p=0.198, **Figure 4A**) but for the competing events aGvHD grade III-IV (p=0.002, **Figure 4B**), indicating that sPD-1<133 pg/ml is an predictor of aGvHD grade III-IV rather than for moderate/severe cGvHD at two months post HSCT. Three months post HSCT a sPD-1 cut-off level of 107 pg/ml (**Table 2**) was exclusively associated with the onset of moderate/severe cGvHD (p=0.011) by univariate competing risk analysis (**Figures 4C, D**). Taken together, low sPD-1 levels two or three months post HSCT are indicators for severe aGvHD or the onset of moderate/severe cGvHD.

### Low sPD-1 Status at Month One or Two Post HSCT Is a Prognostic Co-Variate for Inferior OS, DFS, and TRM

**Figure 5:**
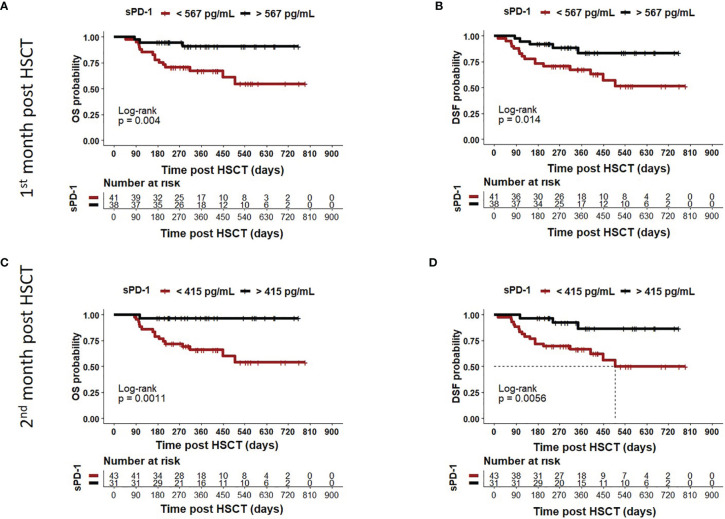
Association of low sPD-1 levels with reduced OS and DFS. Patients were divided in two groups according to cut-off levels obtained one **(A, B)** month (< or >567 pg/ml) and two **(C, D)** months (< or >415 pg/ml) post HSCT. The corresponding Kaplan-Meier curve of overall survival (OS) or disease-free survival (DFS) probability combined with Log-rank test with respect to sPD-1 cut-offs are shown. Patients with sPD-1 below the thresholds in brown showed reduced OS (p=0.004 or p=0.011) and DFS (p=0.0014 or p=0.0056) compared with patients with sPD-1 levels above these values. Dotted line indicates median DFS post HSCT in **(D)**. Due to death or loss of follow up, data were not available for all 82 patients.

**Figure 6:**
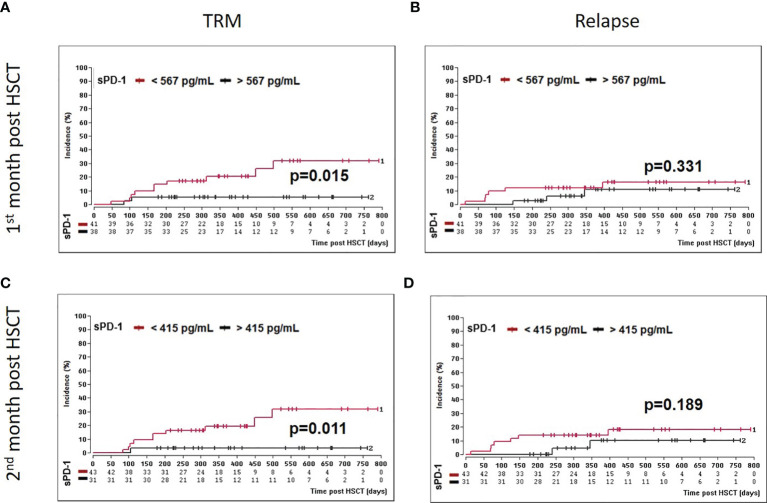
Association of low sPD-1 levels with reduced TRM. Patients were divided in two groups according to cut-off levels obtained one **(A, B)** month (< or >567 pg/ml) and two **(C, D)** months (< or >415 pg/ml) post HSCT. Estimated cumulative incidence curves of patients with TRM **(A, C)** and relapse **(B, C)** as competing event are shown for patients with sPD-L1 < 567 pg/mL **(A, B)** or <415 pg/ml **(C, D)** in brown and compared to patients with sPD-1 >567 pg/mL **(A, B)** or >415 pg/ml **(C, D)** in black.

Moreover, we asked whether sPD-1 plasma levels were associated with OS, DFS, and TRM. A cut-off level of sPD-1 < or > 567pg/ml obtained one month post HSCT (**Table 2**) was significantly associated with OS (p=0.045), whereas this threshold did not reach significance for DFS (p=0.100) and TRM (p=0.056). Kaplan-Meier probabilities of OS (p=0.004; log-rank HR: 5.11, 95% CI: 2.03 - 12.88) and DFS (p=0.014; log-rank HR: 3.29, 95% CI: 1.40 - 7.74) were significantly reduced for patients below this threshold value compared with patients above this level (**Figures 5A, B**). The univariate competing risk analysis for TRM with relapse as competing event revealed statistically different cumulative incidence functions for TRM (p=0.015, **Figure 6A**) but not for relapse (p=0.331, **Figure 6B**).

For the second month post HSCT, a sPD-1 threshold level of 415 pg/ml was identified for OS (p=0.008), DFS (p=0.030), and TRM (p=0.020) by ROC analysis (**Table 2**). Patients with sPD-1 values below 415 pg/ml showed inferior OS probability (p=0.001, log-rank HR: 13.13, 95% CI: 5.05 - 34.14) and inferior DFS (p=0.006; log-rank HR: 4.82, 95% CI: 2.10 – 11.02) with a median DFS of 498 days post HSCT as compared with patients above this cut-off (**Figures 5C, D**). Again, competing risk analysis showed different cumulative incidence functions for patients with sPD-1 < or > 415 pg/ml (p=0.011, **Figure 6C**) for TRM but not for the competing event relapse (p=0.189, **Figure 6D**).

### sPD-1 Levels Are Significantly Increased in ATG-Treated Patients Post HSCT

**Figure 7:**
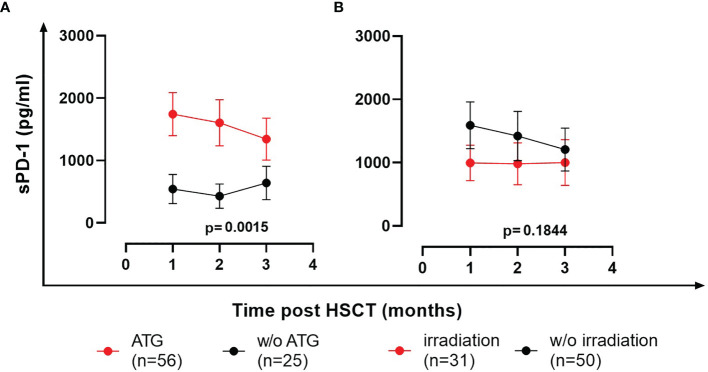
Effects of ATG **(A)** and total body irradiation **(B)** on the course of sPD-1 levels post HSCT. Data are presented as mean ± SEM. sPD-1 levels of treated and untreated (w/o) patients are shown in red and black, respectively. One ATG-patient died within the first month post HSCT and hence was not included in the analysis.

To study the impact of conditioning regimens, patients were stratified into groups of ATG-treated (n=56) and ATG-untreated patients (n=25; one ATG-patient died within the first month post HSCT and hence was not included in the analysis). The sPD-1 levels were nearly 3-fold increased in ATG-treated patients in the first 3 months post HSCT compared to ATG-untreated patients (p=0.0015, **Figure 7A**). In contrast to ATG-treatment, total body irradiation during conditioning did not substantially impact (p=0.1844) the course of sPD-1 levels post HSCT (**Figure 7B**).

### Multivariate Analyses Identifies Low sPD-1 Levels During the First Three Months Post HSCT as Independent Indicators for Moderate/Severe GvHD, Reduced OS and DFS and Increased TRM

**Figure 8:**
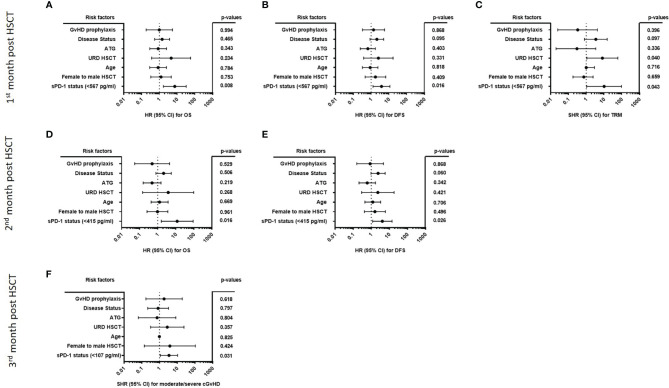
Forest plot of risk factors for moderate/severe cGvHD, OS, DFS, and TRM post HSCT. The forest plots visualize the multivariate analyses of the following parameters for OS **(A, D)**, DFS **(B, E)** TRM with relapse as competing event **(C)** and moderate/severe cGvHD with aGvHD grade III-IV as competing event **(F)**: disease status (early/intermediate versus late), ATG-treatment (yes *versus* no), age at time of HSCT, URD-HSCT (yes *versus* no), female donor to male patient HSCT (yes *versus* no), GvHD prophylaxis (CSA & MTX *versus* CNI & MMF), and the sPD-1 status using the different cut-off levels. For OS, DFS, and TRM a cut-off of sPD-1 < 567 pg/ml of one month **(A–C)**, and a cut-off of sPD-1< 415 pg/ml two months post HSCT **(D, E)** were used, while for moderate/severe cGvHD a cut-off of sPD-1 < 107 pg/ml three months post HSCT was used. Due to death or loss of follow up, data were available for 74 **(A–C)**, 69 **(D, E)**, and 67 **(F)** out of 82 patients; 95% CI, 95% confidence interval; HR, hazard ratio; SHR, Subhazard ratio.

All multivariate analyses encompassed disease status, ATG-treatment, age at time of HSCT, unrelated *vs.* related donor, female donor to male patient, GvHD prophylaxis (cyclosporine A [CSA] & methotrexate [MTX] versus calcineurin inhibitors (CNI) [either CSA or Tacrolimus] & mycophenolate mofetil [MMF]), and the sPD-1 status using the different cut-off levels for moderate/severe cGvHD and OS, DFS, TRM (**Table 2**), respectively, obtained during the observation period of three months post HSCT as co-variates. For OS and DFS, multivariate Cox regression revealed that the sPD-1 status with cut-off values of 567 pg/ml at one month was an independent indicator for OS (p=0.008, HR: 7.42, 95% CI: 1.69 - 32.48) and DFS (p=0.016, HR: 3.88, 95% CI: 1.29 - 11.63) post HSCT (**Figures 8A, B**). Similary, a cut-off value of sPD-1 of 415 pg/mL at two months post HSCT (**Figures 8D, E**) was an independent predictor for OS (p=0.016, HR: 11.99, 95% CI: 1.58 – 91.00) and for DFS (p=0.026, HR: 4.11, 95% CI: 0.96 – 5.91). Concerning TRM with relapse as competing event a sPD-1 <567 pg/ml one month post HSCT was found to be a significant independent indicator (p=0.043, SHR: 2.35, 95% CI: -0.12 – 4.59; **Figure 8C**), whereas sPD-1 <415pg/ml two months post HSCT did not reach significance (p=0.061, SHR: 2.08, 95% CI: -0.09 – 4.25; data not shown). Competing risk regression analysis revealed that a cut-off level of sPD-1 <107 pg/ml at month three post HSCT was an independent predictor for the onset of moderate/severe cGvHD (p=0.031, Subhazard ratio (SHR): 3.45, 95% CI: 1.12 – 10.64) with aGvHD grade III-IV as competing event (**Figure 8F**).

## Discussion

The two most important complications after HSCT are relapse of the underlying hematological disease and graft-versus-host disease. Both complications are mainly determined by immunological processes: Loss of the desired anti-malignancy potency of donor-derived immune cells is one of the major reasons for relapse. In contrast, severe GvHD is induced by an excessive alloreactivity of donor-derived immune cells against patient’s healthy tissue. Thus, a sound homeostasis of immune activity and immune tolerance is essential for the success of HSCT.

In recent years, immune checkpoints as physiological mediators of immune regulation have gained increasing attention. We here present data of *soluble* PD-1 levels after HSCT and its association with the major relevant outcomes after HSCT. In summary, we observed significantly increased levels of sPD-1 in the plasma of patients after HSCT compared to healthy controls and to the pre-HSCT levels. In the plasma of patients with severe acute GvHD grade III-IV, of patients with moderate/severe chronic GvHD, of patients with inferior OS and DFS, as well as with increased TRM, sPD-1 levels were substantially decreased. A sPD-1 status <133 pg/ml at month two after HSCT was associated with aGvHD grade III-IV, and at month three a sPD-1 status <107 pg/ml was found to be exclusively associated with moderate/severe cGvHD in uni- and multivariate analysis. Levels of sPD-1 below the cut-off values defined by ROC analysis for month one and two post HSCT indicated a significantly reduced OS and DSF probability and an increased TRM. Regarding PD-1 prediction of cGvHD associated mortality independently of aGvHD, our data do not support the assumption that the association of cGvHD associated mortality can be viewed independently from aGvHD, since in most cases severe manifestations of cGvHD develop out of severe cases of aGvHD. These are rather continuous manifestations than independent states. This is also supported by the fact that there is no difference in sPD-1 levels between moderate/severe cGvHD cases of quiescent *vs.* progressive type (**Supplementary Figure 2**). In multivariate analysis, low sPD-1 levels in plasma samples at month one or two after HSCT were confirmed as an independent predictive marker for reduced OS and DFS or increased TRM. Finally, in our cohort sPD-1 plasma levels were nearly 3-fold increased in ATG-treated patients in the first three months post HSCT compared to ATG-untreated patients. Notably, no difference was detected among plasma samples of patients treated with or without total body irradiation.

The published data on soluble PD-1 and its interaction with PD-1 expressed on cell surfaces is scarce and has not been investigated in the setting of post-HSCT. sPD-1 is mainly thought to be produced by proteolytic cleavage of membrane-bound PD-1. Specifically, four different splice variants have been described while only one (PD-1Deltaex3) is likely encoding the soluble form of PD-1 (16). Increased levels of soluble PD-1 have been associated with advanced disease stage and – although not consistently – with worse prognosis in several malignancies (reviewed in (10)). Indeed, in this study a high variability of sPD-1 levels was observed for healthy controls and for pre/post HSCT patients. Furthermore, a wide range of cut-off values was defined for aGvHd/cGvHD and OS/DFS/TRM. Thus, further investigation are needed to clarify mechanisms of sPD-1 release into circulation and its functional consequences in view of aGvHD/cGvHD and OS/DFS/TRM post HSCT.

This study analyzes the course of sPD-1 levels prior to allogeneic HSCT and in the first three months after HSCT. Despite being a prospective study, it has several limitations. Firstly, the results of this mono-centric patient cohort of 82 patients need to be validated in larger and also multi-center studies. As we were able to show an inverse correlation between PD-1 expression on T cells and sPD-1 levels in peripheral blood only for a limited number of patients post HSCT, the relationship of membrane-bound and soluble PD1 has to be further elucidated especially in view of the functional and clinical relevance for HSCT outcome. Secondly, the influence of medication, notably immunosuppressive agents, and of infections on the course of membrane-bound and soluble immune checkpoints and their interactions have to be studied. Finally, the functional role of sPD-1 for the clinical HSCT outcome remains to be further elucidated. Interestingly, it has been reported that sPD-1 molecules are able to inhibit dendritic cell (DC) mediated CD4+ T cell activation and Th1 and Th2 cytokine production *via* the interaction of sPD-1 with PD-L1/2 expressed on DC, which results in a reduced expression of maturation marker and a suppressive DC phenotype (17).

In conclusion, the results presented here provide substantial evidence that soluble PD-1 could be a promising novel biomarker predicting severe GvHD and inferior survival after HSCT and opens up the discussion about the functional role of sPD-1 molecules in HSCT. In addition, we observed the influence of conditioning on the plasma levels of sPD-1, as sPD-1 plasma levels were nearly 3-fold increased in ATG-treated patients. Hence, further studies could help to evaluate the influence of immunosuppression and infections on sPD-1 levels and refine conditioning regimens to further improve HSCT outcome.

## Data Availability Statement

The raw data supporting the conclusions of this article will be made available by the authors, without undue reservation.

## Ethics Statement

The studies involving human participants were reviewed and approved by Ethical Board of the University Hospital of Essen, Germany (07-3503). The patients/participants provided their written informed consent to participate in this study.

## Author Contributions 

VR and LK conceived, designed and performed the experiments, analyzed the data and wrote the manuscript. UB, FH, BG, PH, DB, and HR confirmed the analyses and assisted in correcting the manuscript. All authors contributed to the article and approved the submitted version.

## Funding

This study was supported by the European Union and the German federal state North Rhine-Westphalia (“SEVRIT” project, EFRE-0800396).

## Conflict of Interest

The authors declare that the research was conducted in the absence of any commercial or financial relationships that could be construed as a potential conflict of interest.

## Publisher’s Note

All claims expressed in this article are solely those of the authors and do not necessarily represent those of their affiliated organizations, or those of the publisher, the editors and the reviewers. Any product that may be evaluated in this article, or claim that may be made by its manufacturer, is not guaranteed or endorsed by the publisher.

## Supplementary Material

Supplementary Figure 1 No association of pre-sPD-1 levels with gender and disease type. sPD-1 levels are shown by Box-Whisker plot with minimum and maximum.

Supplementary Figure 2 Course of sPD-1 levels in patients with progressive and quiescent type of moderate /severe cGvHD. Due to the low number of patients (n=1), data for sPD-1 levels with de novo type of moderate/severe cGvHD were not shown.

Supplementary Figure 3 Association of low sPD-1 level with moderate/severe cGvHD and aGvHD grade III-IV at month one post HSCT. Patients were divided into two groups according to cut-off levels (< or >461 pg/ml) obtained one month post HSCT. Estimated cumulative incidence curves of patients with moderate/severe cGvHD **(A)** and aGvHD grade III-IV **(B)** as competing event are shown for patients with sPD-1 <461 pg/mL in brown and compared to patients with >461 pg/mL in black. The sPD-1 levels were available only for 73 out of 82 patients due to death or loss of follow up.
