# Supplementary figures and images for: Low Soluble Programmed Cell Death Protein 1 Levels After Allogeneic Stem Cell Transplantation Predict Moderate or Severe Chronic GvHD and Inferior Overall Survival
Source: Front Immunol. 2021 Sep 24;12:694843. doi: 10.3389/fimmu.2021.694843 (PMC8498033; doi:10.3389/fimmu.2021.694843)

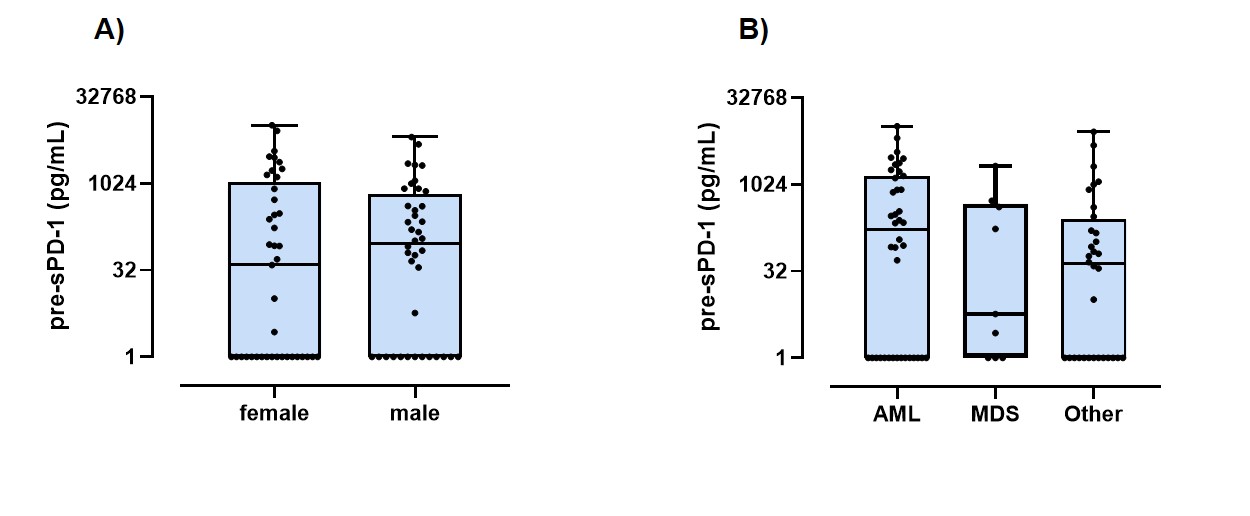

Supplement: Supplementary Figure 1 — No association of pre-sPD-1 levels with gender and disease type. sPD-1 levels are shown by Box-Whisker plot with minimum and maximum. [file Image_1.jpeg]

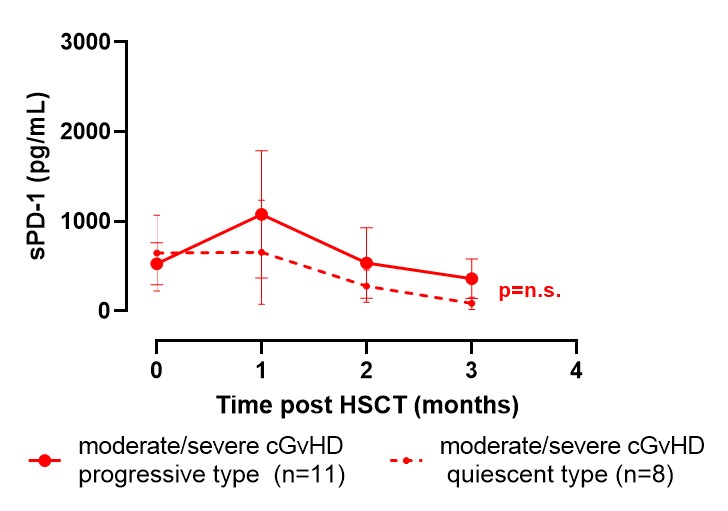

Supplement: Supplementary Figure 2 — Course of sPD-1 levels in patients with progressive and quiescent type of moderate /severe cGvHD. Due to the low number of patients (n=1), data for sPD-1 levels with de novo type of moderate/severe cGvHD were not shown. [file Image_2.jpeg]

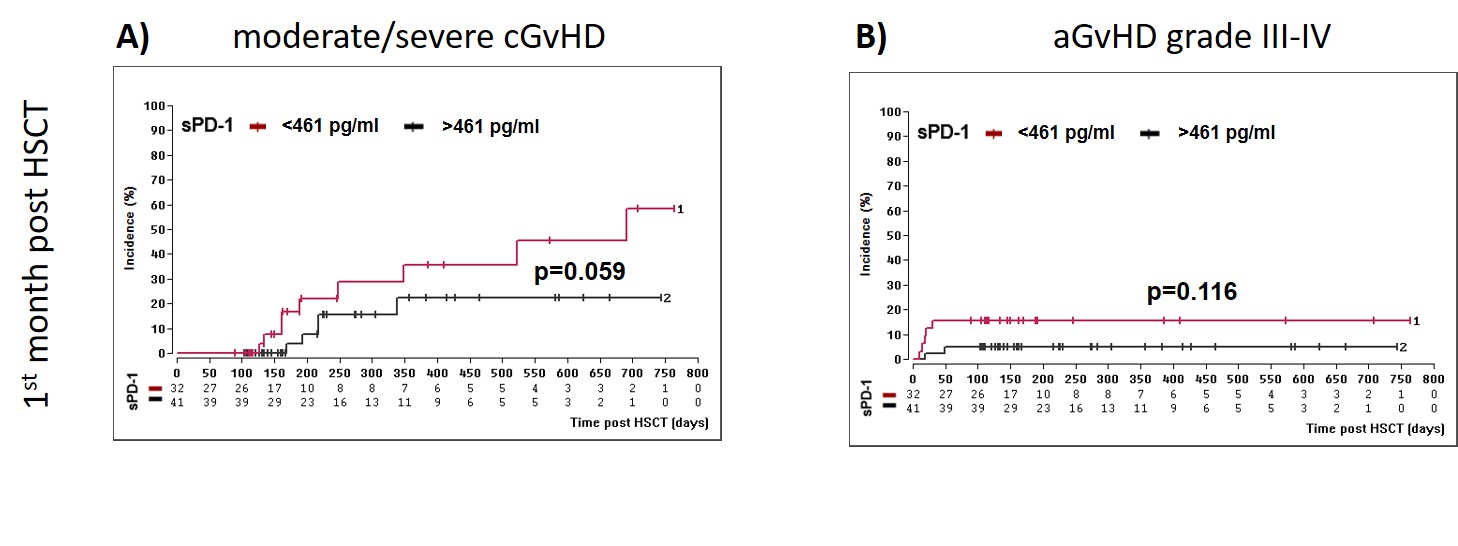

Supplement: Supplementary Figure 3 — Association of low sPD-1 level with moderate/severe cGvHD and aGvHD grade III-IV at month one post HSCT. Patients were divided into two groups according to cut-off levels (< or >461 pg/ml) obtained one month post HSCT. Estimated cumulative incidence curves of patients with moderate/severe cGvHD (A) and aGvHD grade III-IV (B) as competing event are shown for patients with sPD-1 <461 pg/mL in brown and compared to patients with >461 pg/mL in black. The sPD-1 levels were available only for 73 out of 82 patients due to death or loss of follow up. [file Image_3.jpeg]
